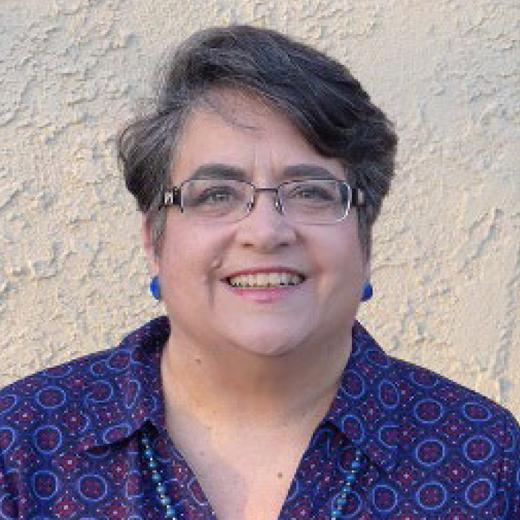# Building an Equitable Future Through Data Disaggregation

**DOI:** 10.1089/heq.2023.29036.rtd

**Published:** 2023-04-21

**Authors:** Tina J. Kauh, Terry Ao Minnis, Meeta Anand, Maya Berry, Rosalind Gold

**Affiliations:** ^1^ Senior Program Officer, Robert Wood Johnson Foundation, Princeton, New Jersey, USA.; ^2^ Senior Director of Census and Voting Programs, Asian Americans Advancing Justice (AAJC), Washington, District of Columbia, USA.; ^3^ Senior Program Director for Census and Data Equity, The Leadership Conference on Civil and Human Rights and The Leadership Conference Education Fund, New York, New York, USA.; ^4^ Executive Director, Arab American Institute, Washington, District of Columbia, USA.; ^5^ Chief Public Policy Officer, NALEO Educational Fund, Monterey Park, California, USA.

***Dr. Kauh:* Race and ethnicity data are so critical for understanding the health and well-being of everyone living in our nation. We are in the midst of an exciting time, where there is a major opportunity to actually impact how race and ethnicity data are collected, analyzed, reported, and disseminated to promote greater health equity.**

**As we all know, the Office of Management and Budget, which is also known as the OMB, is considering for the first time in >25 years whether to update its current minimum standards for race and ethnicity. I want to keep saying that word, minimum standards, because I think that is really critical. I will start by asking Meeta, can you explain why this effort by the OMB to revise federal race and ethnicity minimum standards is so important?**

**Ms. Anand:** So, you already touched on part of it. The last time that OMB issued new guidelines on the collection of race and ethnicity data at the federal government level was in 1997. Our perception of who we are, both as a nation and as individuals, has shifted since that time. But on top of that, what we also see is that it is not just, when we say, is the federal government that is collecting the data, think about what that means and how that trickles down. Anyone reporting to the federal government is adhering to those standards. It means that people often default to those standards. Because they think someone else went and spent time and put effort behind this, and these must be the right categories to use.

So, what this really represents to us is an opportunity to make sure that certain communities who feel that they are not seen or reflected in the data can then start being seen or reflected in the data by the categories that are provided for people to choose among and being able to self-identify. Why does this matter? Because all of us here are deeply committed to understanding and remedying inequities we see in our society that exist on racial and ethnic lines. They exist on other lines too, but we are focusing on racial and ethnic lines. If we are able to have more accurate data that are presented on the basis of race and ethnicity, we are then able to more accurately identify inequities, and then hopefully, target inequities in our policy and programmatic practices.

***Dr. Kauh:* Thanks so much, Meeta. Also, one follow-up question. Oftentimes, when we talk about race and ethnicity data, the phrase disaggregated data comes up. Can you just explain what that term means?**

**Ms. Anand:** Absolutely. So, I personally define it as whatever you are collecting, it is one step further in collecting race and ethnicity data. So, what do I mean by that? So, if you are collecting data, say, and you have a category that is Asian as a race, disaggregated data would allow you to pick Chinese or Bangladeshi. So, it is always that one level more of granularity in race and ethnicity data that allows people to more specifically identify what they view to be their race or ethnicity.

***Dr. Kauh:* Thanks, Meeta. And Maya, can you talk a little bit about, why is data disaggregation essential to promoting health equity?**

**Ms. Berry:** I think, for the very reasons Meeta just lead with. When we talk about health equity, there are individual risk factors that I might have personally, whether it is my genetics or my own behavior. But there are also a whole other set of categories that come into play in terms of the social determinants of health. We talk about language access. We talk about poverty levels or income. We can talk about insurance coverage. We can talk about discrimination. But access to care to begin with is sometimes impacted by these very, very real issues.

How do we gather information about some of those issues? It all comes back to our ability to capture information about communities in the data. If a hospital is operating in a community but it is not aware that there has been a recent influx of immigrants and refugees who may speak Arabic, for example, in that region, they may not have someone on staff who can provide the translation services that are needed. That is one of the countless ways in which all of this plays out. The disaggregate piece is a critical part of getting to that.

***Dr. Kauh:* Terry, can you talk about why this is such an important issue for, specifically, civil and human rights?**

**Mrs. Minnis:** The collection of accurate race and ethnicity data is critical to obtaining an accurate portrait of an increasingly diverse U.S. population and to revealing and addressing inequalities, as others have mentioned. More accurate race and ethnicity data allow us to understand the share of resources that our communities are receiving and are essential for the implementation and enforcement of civil rights laws, including the U.S. Constitution's guarantee of equal representation. To further civil rights, it is important for the collection of race and ethnicity data to include the collection of detailed disaggregated data, as Meeta had mentioned.

Broad racial and ethnic categories do not allow for a full true understanding of our populations and can disguise underlying trends that can illuminate needed policy remedies that federal agencies really must be required to collect detailed race and ethnicity data. Although the current OMB standards allow for the collection of detailed race and ethnicity data, what we have seen is that agencies have treated the current standard as a ceiling and not a floor and have not proactively engaged in data disaggregation. This has been a big problem for many communities, including Asian Americans, Native Hawaiian, and Pacific Islanders.

For example, Asian Americans are made up of >30 countries of origin, 50 ethnic groups, and speak >100 languages. And as one of the fastest growing major racial and ethnic group over the decades and with a diverse history of colonization imperialism and migration, Asian American groups today have wide-ranging differences across almost all socioeconomic characteristics, as Maya mentioned. Without accurate data by detailed groups, some of the most disadvantaged in our communities are rendered invisible to policymakers, leaving their critical needs unmet. I will just give a quick example around the COVID-19 pandemic.

We know that the pandemic exposed disparities and unaddressed systemic issues for many communities. And for Asian Americans and Native Hawaiian Pacific Islander communities, COVID-19 was just another stark reminder that data disaggregation can be a matter of life or death. Again, not specific just to our community, this is true for all communities. But it was interesting to see that there were a couple of data collection issues around the pandemic, including some states who just failed to provide data on Asian Americans or Native Hawaiian Pacific Islanders. We also had other states that aggregated the two racial groups, thereby making invisible the larger risk that the Native Hawaiian Pacific Islander community often saw. I will just give a couple of examples.

In California, when you looked at the death rates per 100,000 people in 2020, the state total was 84. Combined Asian and Native Hawaiian Pacific Islander death rate was at 75. But for Native Hawaiian Pacific Islanders only, it was at 123. Then when you look at the data disaggregation, as Meeta was talking about, then looking at the Samoan and Tongan population, their death rates were even higher than the Native Hawaiian Pacific Islander rate, at a rate of 182 and 124, respectively. Similarly, in Wisconsin, we saw the Hmong population had a disproportionate health impact of COVID with respect to the number of cases, hospitalizations, and death.

It is important because if you do not understand the communities that are being affected the most, then the plans that you develop will not address their needs. They will not be culturally and linguistically appropriate. They will not reach the audience that they are meant to reach. It is important that we have this ability to require agencies to disaggregate data. I like to say, disaggregating data allows us to aggregate up to the larger community, but an aggregate data point alone does not allow us to understand the makeup or foundation of that data.

***Dr. Kauh:* Thanks, Terry. The examples you provided were so striking and really make the case for why it is so critical. Currently, the OMB requires that race and ethnicity are collected using two separate questions. One of the proposed changes is actually combining the race and ethnicity questions into a single item. Rosalind, could you talk a little bit about how that change would affect the Latino community?**

**Ms. Gold:** I would like to start by saying that we are completely in agreement with all of our colleagues that accurate data on race and ethnicity are critical to the health of our democracy, the health of our society, and the health conditions of all our communities, especially for examining and identifying different health outcomes and health disparities. With respect to the Latino community, revising the OMB standards to allow a combined question on race and ethnicity is critical for the collection of complete and accurate data on the Latino community and all of our nation's population. Extensive research by the Census Bureau, as well as our own experience with the Latino community, really demonstrate the problems with the existing two question approach.

The research by the Bureau shows there has been an evolving mismatch between the racial categories in the Census race question and how Latinos report their self-identification. Many Latinos simply do not see themselves in the race categories in the race question. They either skip it or indicate that they are of “Some other Race.” The research by the Bureau has included focus groups, interviews, and an extensive study, the 2015 National Content Test, which is the largest field test conducted by the Bureau. It included 1.2 million households and oversampled census tracts with Latinos and other population groups.

In this research, the Bureau found that many Latinos embrace their Latino identity as their sole identification. In some cases, Latinos were indicating they were White, because they felt they had to provide an answer to the race question. Census 2010 and 2020 data also show the problems with this approach. In both censuses, nearly half (44%) of Latinos indicated they were of Some Other Race or skipped the race question. In Census 2020, Some Other Race became the second largest racial group in the country after White.

The large number of Latinos who either skip the race question or indicate Some Other Race also presents problems for data consistency between census data and other federal data. As noted, the census question on race includes the Some Other Race category, but this is not a category in the OMB standards. Thus, to achieve consistency between decennial census data and other federal data sets, including the Bureau's own population estimates, the Bureau basically assigns a race to Latinos who skip the race question or indicate Some Other Race. As a result, many Latinos are assigned to a race group with which they do not identify. Moreover, because of the statistical approach used by the Bureau to make this assignment, most Latinos are assigned to the White category. So, for many data sets, the population looks more White than it actually is. This is another serious problem that could have implications for data that are examined regarding health outcomes.

Ultimately, the Bureau's research shows that the combined question approach best aligns with how Latinos identify. The nonresponse rate to the combined question is significantly reduced. And <1% of Latinos indicate that they are of Some Other Race. The question also actually increases the share of Blacks who also identify as Latino. And there is no reduction of the number of Latinos who identify as both Latino and American Indian and Alaska Native. Finally, the combined question allows the collection of data on Latinos who identify with more than one national origin or subgroup. For example, instead of being able to identify solely as Salvadoran or solely as Dominican, respondents can indicate that they identify as both Puerto Rican and Dominican, or as Salvadoran and Mexican, which is another way to achieve better disaggregation. All of these improvements would allow the collection of data that could be used to better understand the nuances and complexity of health issues for the full diversity of the Latino community.

***Dr. Kauh:* One of the other major changes that the OMB has proposed is adding Middle Eastern or North African, or also known as MENA, as an ethnic category as a response option for a new combined race and ethnicity question. And Maya, I was wondering if you could talk a little bit about how you think those two changes would affect the MENA community.**

**Ms. Berry:** Well, we care, as advocates, about data equity. But the Census Bureau has done extensive testing that now got us to the point of saying, to better collect data about these communities, a combined race and ethnicity question will allow us to do that. It finally addresses what has been happening for decades, which is that the folks from the Middle East and North Africa have been rendered completely invisible in the data. From 1980 to 2000, there was an ancestry question on the long form of the census that was the only place where we could gather any data about our communities. Once we lost that in 2000, we have not had anything other than the American Community Survey.

Although the American Community Survey (ACS) is very important to all of us, there are very real limitations on getting the aggregate data about our community from the ACS. In fact, that is an important point to be made here. Because I feel like my colleagues here can have a conversation about disaggregate data in a way that I cannot, when we talk about MENA populations. Because we are not there yet. We are not even collecting the aggregate data to talk about the need to get it to a detailed subgroup within that. So, for folks from the MENA region, and specifically, I represent Arab Americans who would make up the largest segment of population within MENA, it would be transformative.

It would be for the first time ever a category is added so that our folks can look at the census form, can look at the American Community Survey, find a MENA checkbox, check that. And within that, write their own ethnicity or national origin or race category should they identify with it. And Tina, the way you phrased it I think is important and another historic first. This is the first time we are able to say, we support the recommendations of the initial working group. They have suggested the addition of a MENA minimum reporting category, although they lack the clarity on whether it is an ethnicity or race.

But it is really critical that we talk about it as an ethnic category. It is a diverse region. We are talking about 22 members of the Arab League plus 3 countries that fall within that geography which are Israel, Iran and Turkey. And then within that, we have transnational communities, including Armenians or Assyrians and Chaldeans or Kurds. It is a wonderfully diverse part of the world that is represented here in the United States in a very real and meaningful way.

It is critical that it be an ethnic category, so people are able to identify both their ethnicity and the racial category that they may identify with. The Census Bureau found that, when they included a MENA a category, that it actually improved the counts for other communities as well. We saw an increase in the Afro Arab identification. We saw an increase in the Arab Latino community. So, when you have detailed questions and the ability to find yourself on a form, you invest in that form. It is a signaling bias. It does, I think, allow folks to self-identify in a way that is more representative of who they are.

I love data. They are important. They are critical. But without a combined question and this change in these race and ethnicity standards that has not happened in more than two decades, my community would be rendered invisible and then harmed for at least another decade by the lack of data.

***Dr. Kauh:* Both of you touched on this idea of how critical it is for individuals to be able to see themselves in the options, that they have the ability to self-identify within these proposed updates to the OMB minimum standards. Meeta touched on this in her opening remarks, about the fact that when we talk about the OMB's minimum standards, we are talking about federal standards. But they have implications for state and local data as well. What do you think is needed to support state and local health departments in implementing new race and ethnicity standards and ensuring broad implementation?**

**Ms. Anand:** At the Leadership Conference, with the help of the Robert Wood Johnson Foundation, we have stood up our data disaggregation network, which targets five states and has local partners in those states, who really are working with communities and with local public officials as to, how do we think about data? How do we engage with data disaggregation? And why is this important?

You have some level of disconnect between the state standards that are applied and the federal standards that are applied. In a lot of instances, just moving the states to the old OMB standards would allow us to have more disaggregation than we do now.

If we are able to see revised standards that we are all hoping for and then move the states to those standards, which would have even more categories of race and ethnicity and intersectionality that we all want to see and we move the states to that as well, then we would be able to achieve the dream of being able to see our communities and understand the differences. So, I think the first step is really education of communities and education of our local and state officials and public agencies within those communities, those states, about why this matters and the fact that there are standards out there that have been thought about that, if they just move to that, it will allow for an easier process for them going forward.

**Mrs. Minnis:** I am going to start where Meeta left off around helping the states move to even the current standards. Again, the current standards allow for data disaggregation, which, unfortunately, as I mentioned, has not actually resulted in more agencies doing it. But I think it is important that there is a standard out there that advocates and community-based organizations (CBOs) can point to, to say, no, there are precedents within agencies to do this.

In that same vein, that is one of the reasons why the initial proposed revisions as contemplated by the Federal Register notice to move that disaggregation data collection and reporting to a mandatory nature as opposed to a permissive nature is also important. Because it is a strong statement, again, to point to for advocates to be able to say, no, state and local, you should be doing this as well.

To that point, I would also say that what would be helpful is to have strong guidance and language from OMB. They currently have a guidance that they issued earlier last year that lifted up the idea that, under the current standards, you can disaggregate data. So, I would start there as something for today. Then, as we move along in the process and hopefully see revised standards that move to mandatory, there will be strong guidance from OMB about that as well.

We can continue to lift that up as model language for state and locals and to help with the education process that Meeta was talking about. Also, there are some places that have started to do this work already. California has done some work. New York. Look to those as potential models or at least lessons learned, things that worked well, maybe some things that did not. You can learn from what the Census Bureau has done in their data collection processes.

It will be important to have strong guidance and technical assistance at the state level. So, taking that information from other states, from the federal government, but also ensuring that, at the state and local level, that there is strong guidance from the state. Consistency is key. Being able to have comparable data across agencies is key.

The only way you get there is to have everybody working off of the same playbook, so that they are not asking the same question in five different ways, so that the answer is slightly different. The last thing I would just mention, CBOs must be engaged. They really are the experts on their own communities. Utilizing them to help check for whether the right examples are being used, ones that actually resonate in the community? And frankly, they are also your trusted messengers. They can also help make sure that people who have questions have a resource that they can go to in trying to figure out what is being asked and how to best answer it.

**Ms. Gold:** When we think about something as fundamental as changing the OMB data standards to move from a two separate question approach to a combined question approach, we are not going to see the improvements in data completeness and accuracy unless we implement this in a very, very sound manner. There are several issues regarding implementation that are particularly important for the Latino community. I also want to acknowledge that many of the concerns that I bring up are partly informed by the fact that there are Afro-Latino researchers and civic leaders who do not support the adoption of the combined question. We believe they raise very serious and important issues, and that the implementation of the combined question for the Latino community should be shaped by many of the concerns we have heard.

First of all, I want to lift up something that Maya mentioned, which are the instructions, the wording, and the format of the overall combined question. These need to really be clear about the distinction between race and ethnicity. The combined question also must clearly indicate that people should check all minimum categories that apply. With respect to the Black category, it is very important that the checkboxes and the examples used for that category clearly signal that Afro-Latinos should check that box if that is how they so identify.

In addition, another important issue for implementation is how to soundly tabulate data. How do you make it accessible to people? This must be done in a way where groups can get easy access to data at disaggregated levels, such as Afro-Latinos or Latinos who identify as Latino and another ethnic or racial category. Finally, the OMB and the Census Bureau must give guidance to localities and states on how to implement this in the foregoing ways. This guidance should include the need for an opportunity for agencies at all levels of government to do more nuanced and disaggregated analysis.

Finally, I very much agree with Terry's point that none of this can be accomplished in a sound way without close consultation with the full diversity of stakeholders in the Latino community. The OMB and the Bureau should work closely with diverse Latino stakeholders in determining how to do outreach about the combined question, including which are the best messages and who are the best messengers to explain the implications of the new question. This also means that any research that the Bureau does needs to have very robust and representative samples of Afro-Latinos and the full geographic racial and ethnic diversity of the Latino community.

**Ms. Berry:** These are significant changes that are going to be made in terms of combining the race and ethnicity question and specifically in terms of adding a new MENA category. I think we have to be sensitive to how long this process will take and the resources that are going to be necessary to get there.

The other point I would make is that, although this is happening on the federal level with regard to census and federal data collection, there are examples in states across the country where local health departments are actually ahead of the federal level on this issue, particularly with regard to collecting information on the MENA populations. I would point to the State of Michigan, for example, where when we talk about being rendered invisible in the data and the importance of public health data needing to respond more adequately.

We know, for example, there is a prevalence of diabetes in the Arab American community. We do not have the information specifically to tell us how much. What we can do is there have been certain ethnic enclaves across the country that have gone in and done some very specific research on their local communities. Michigan has been the example of that for many, many years. I am from Michigan, the highest concentration of Arab Americans in the entire country. That is not the same thing as, for example, Peoria, Illinois, which had a historically Arab American community there for generations.

It is very different than Paterson, New Jersey, or Orange County. You cannot take the ethnic enclave data and then extrapolate it nationally. But it is very heartening to see that those who have been on the local level and those state health departments that have sought to improve the health outcomes for their communities, they have been ahead of this. When the State of Michigan told us that Arab Americans were 2.63% more likely to test positive for COVID than non-Hispanic Whites, it is because of the research that they did in those areas. And how did they get that? Well, they used ACS data with all of its limitations when it comes to our folks. They used surname lists.

We have literally been running these hacks to get to a place of being able to pull this information and to produce what is necessary in these local communities so that the health outcomes can be addressed and improved for people. So, I am thrilled it is happening on the federal level. I think it is going to be difficult to implement on the local level. But we can certainly do it, and it is important to do. But I also think there are a lot of examples across the country where we can learn from what is happening at the state level up.

**Ms. Anand:** If I may, Tina, I would love to interject two more points that I should have raised earlier. One is we are, as everyone has noted, in the comment phase for the OMB race and ethnicity standards. One thing we have been doing is encouraging state groups, such as state CBOs, to get involved in the comments. And one comment they could include is what they think is necessary for the implementation and what role the federal government can play in that.

There are always sensitivities between and among jurisdictions. But it is always possible not only to have guidance, but also to convene groups to talk about, what does it look like to implement this at a state level or local level, if you choose to do so? And the other thing I would point out is many states have a chief data officer. So, chief data officers can form a learning network of their own. As Terry put it so beautifully, no one needs to do this alone. No one must reinvent the wheel at this moment.

Creating those learning opportunities and sharing opportunities where, as people try to implement either the revised standards or a version that is more applicable to their community or try to just implement part of OMB's revised standards, as they do that, learn from the others as to how they did it and why they did it. That is also part of the community we are trying to implement here, where you have the national partners working at that national level to see improvements, but really have that learning ability for the state partners to learn from what is happening at the national level but tailor it for the state level.

**Ms. Gold:** I will take this opportunity to raise another issue regarding stakeholder input. I think it is important to hear from governments as well as health data users. For the Latino community, this is especially important with respect to the adoption of the combined question. Because with the adoption of the combined question, we are likely to see that there are a lot of Latinos who are not going to indicate any racial identification beyond their ethnic identification as Latinos.

With the adoption of the combined question for the decennial Census, you would see a dramatic increase of Latinos who do not report any racial category identification. We want to hear from health data users about the implications of this increase for examining disparities or health outcomes within the Latino community. Are there going to be challenges by not having data that we might otherwise have? Now, I should note that, under the two separate question approach, we are not getting that racial data anyway because so many Latinos are marking off some other race. But we really do need to hear from health data users.

***Dr. Kauh:* All of you listed out such important issues and around how we can support both state and local implementation of these standards and getting input from both state and local stakeholders all the way down to individual level stakeholders who are actually using the data. One of the things that I find striking from this conversation is how we have talked about the fact that the last time the standards were revised was in 1997. And we are still struggling to get state and local agencies to adhere to those standards. How we are going to encourage and support state and local agencies to adhere to revised standards in a timely way is going to be so critical. Or else we will still be sitting here 30 years from now still trying to catch up.**

**I am curious about your thoughts on this in the context of data equity. When I look at the proposed changes that the OMB has raised, the third major change has to do with requiring detailed checkboxes under those broad ethnic and racial categories. But the thing that really caught my attention was this caveat that the detailed checkboxes based on the National Content Testing in 2015 seemed to yield better data. So that is why they are recommending it. But there is a caveat that agencies do not need to implement that if they deem the burden of doing it to outweigh the potential benefits. When you think of data equity, it is really about what defines a burden? And who gets to decide that something is a burden? So, I was wondering if any of you have thoughts on that aspect of the proposed changes?**

**Ms. Berry:** Terry made the point already, that these standards are the floor, not the ceiling. So, it is incredibly important that we understand that the detailed collection is part of the objective here. For example, in our case, if you just check MENA, I daresay that does very little for my ability to do my work. The Iranian American community, I trust, would say the same thing. The Armenian American community would do the same. I think it is important that we understand that it is a requirement. But the part that I struggled with when I read the recommendations was, in what universe, in terms of today's contemporary technological advancements, can you possibly make the case that the collection of that subcategory data is such a burden on you that you cannot do it? I mean, the 2020 census included an online form.

So, what is happening out there, in terms of federal agencies, where that additional piece is such a tremendous burden that it cannot be done? I think this is simple cost–benefit analysis. I would argue this is the same when we talk about why state and local communities are going to want to meet these standards. Because at the end of the day, it actually allows them to provide better services to their constituents. So, I think you are going to get buy-in from across the board on this, because it is more helpful. I think they would be hard-pressed to make a case that it is an undue burden and that it is too difficult to do it. We have made some pretty serious advances now, and I think we can do so using technology.

**Mrs. Minnis:** I agree with Maya. And I will also say, even thinking through the different agencies and state and local, I recognize that, much like our communities, it is not a monolith with the government. There are different agencies, different sizes, and different resources. I think that it is not bad to say, let us recognize that. What we will be urging to change is that that decision cannot be unilaterally made by the agency. That is, an agency cannot decide, oh, well, this is too burdensome. So, I am just going to opt out and go with, the minimum category format. It might make sense to say, there is an exemption process that must be adhered to that there is a third party. Maybe it is OMB, maybe somebody else who is a deciding authority as to whether or not an agency could be exempt from having to do data disaggregation.

The burden will then be on the agency to apply for the exemption, to provide the proof as to why, in the cost–benefit analysis, as Maya said, that it is just too burdensome for them to do it. We will be suggesting that the process includes some type of publicizing aspect to ensure that interested stakeholders are made aware when an agency is inquiring or requesting an exemption. And finally, there must be some sort of time frame built in that would allow for the public to provide comments about an agency. In addition to OMB or the third party having an affirmative obligation to reach out to the communities that would be impacted particularly by that agency's decision, and then through that process, there could possibly be an opportunity to get exempted. But it should be not the default, and it should be a high standard for an agency to reach, in large part because of what Maya said, that with technological advances, it is a little bit hard-pressed to understand what the burden may be.

**Ms. Gold:** I want to amplify both the points that Terry and Maya have made. Bottom line, there had better be an extremely compelling reason why an agency—and it should have to demonstrate that—should not provide the detailed data in the standards. But I would also note, look, to get to this point, the OMB has convened a working group of technical folks from all of the different federal agencies. Why not keep this dialogue going? Why not have agencies share best practices with each other?

If an agency is running into an issue, why not use this as a foundation for building a more formal network perhaps? Or maybe even strengthening informal relationships, so that people can maybe do troubleshooting. And what might look like an incredible burden might not look so much like a burden when you get a chance to see how other federal agencies are doing it. Let us get much more collaboration. The OMB has an important leadership role to play in this sense. Not only, like I said, collaboration between the federal agencies, but also providing the technical assistance for state and local agencies that might be experiencing challenges.

**Ms. Anand:** That is just such a perfect point to tee my thought up, which is, next week, the Leadership Conference will be issuing a report called Data for Equity, which will be exploring ways in which federal agencies can further improve their data collection practices and data disaggregation in furtherance of their own equity action plans (https://civilrights.org/resource/data-for-equity-a-review-of-federal-agency-equity-action-plans/). And I bring this up for two reasons. One is the point that we make in the report is exactly around cost–benefit analyses.

You need to take equity into account when you are doing these analyses. So if you are saying, “The cost is high, but the benefit is minimal,” because you are looking at a small area or a small population, then that is actually unduly placing a burden on that small community for the very fact that they are a small community. And that is an inequitable outcome.

The second point is one of our recommendations is exactly what Ros just said, which is that OMB and the subcommittee for equitable data should really serve as a clearinghouse for ideas and best practices. Let us have the government stop working in silos and share with each other the best ways to do this and to collect the data. There is really creative work going on within some of the government agencies, but the other agencies do not know about it. If they are able to share that information, then they will be better able to understand that maybe that burden is not as high as they thought.

**Ms. Berry:** May I come in with a friendly amendment? The data statisticians are incredibly important to this discussion, and we see that repeatedly. So, I am not suggesting that it is one or the other. But at the same time, if you do not have community stakeholders at that table with the data folks, then you will not have, I think, the desired outcome for improving data equity across the board in all areas. As we think about this moving forward, it is critical that the stakeholder organizations, the community representatives be part of that discussion. Because if it is strictly about the statisticians in the room—love them, we need them, but we will not get to where we need to go.

I think that is an important piece of this. The first federal agency to give us a MENA category was United States Agency International Development (USAID). And it was not because USAID was doing external foreign policy. It was because there were specifically people from the MENA region who worked at USAID who brought it to the director's attention that this did not exist. So, we had a group of Arab Americans who came together and helped make it happen at a federal agency before it was part of the OMB's initial working group recommendations. Sometimes things happen that way. And the agency of those individuals is important in the process.

***Dr. Kauh:* Thanks so much to all of you. I think this was such a fabulous way to end the conversation. I think all of you lifted up the fact that, although potentially impacting OMB minimum standards is a huge opportunity, it is really only the first step in a very long process. And it is going to involve engagement from individuals and organizations at all levels, from local, state, and federal, as well as individuals who are interested in using the data themselves to those who are analyzing and reporting it.**

## Figures and Tables

**Figure:**
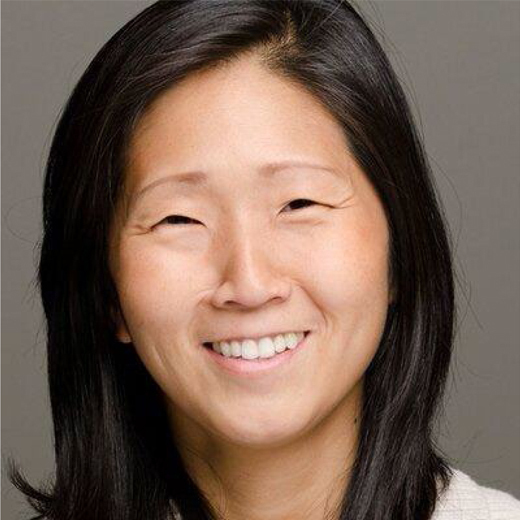


**Figure:**
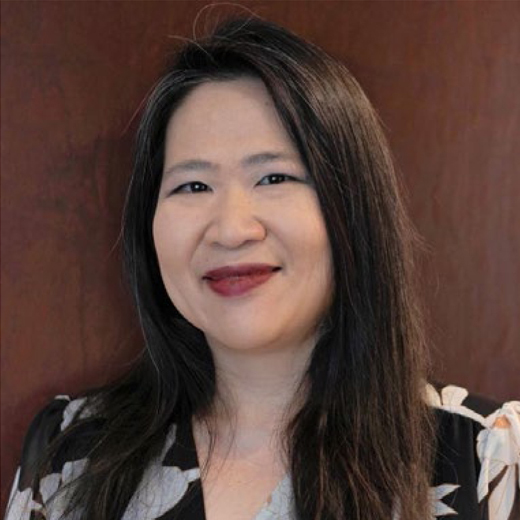


**Figure:**
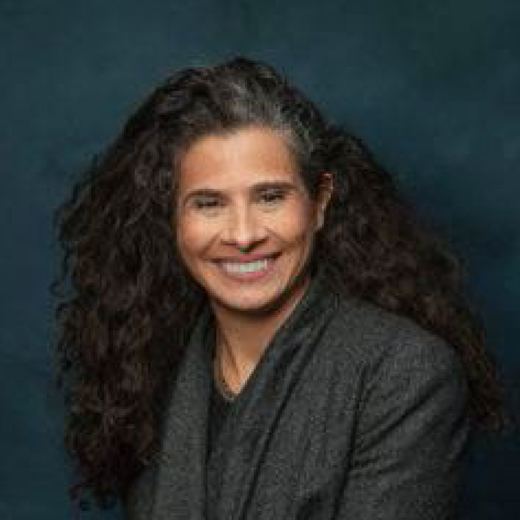


**Figure:**
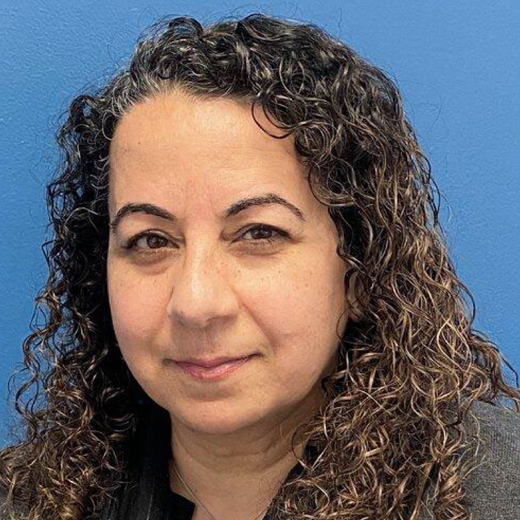


**Figure:**